# Synthesis and Thermoelectric Properties of Pd-Doped ZrCoBi Half-Heusler Compounds

**DOI:** 10.3390/ma11050728

**Published:** 2018-05-04

**Authors:** Degang Zhao, Min Zuo, Lin Bo, Yongpeng Wang

**Affiliations:** School of Materials Science and Engineering, University of Jinan, Jinan 250022, China; mse_zuom@yeah.net (M.Z.); mse_bolin@yeah.net (L.B.); mse_yongp@sina.com (Y.W.)

**Keywords:** ZrCoBi, half-Heusler, Pd/Co substitution, thermoelectrics

## Abstract

In this study, *n*-type Pd-doped ZrCo_1-x_Pd_x_Bi (*x* = 0, 0.03, 0.06, 0.09) half-Heusler samples were prepared by arc-melting and rapid hot-pressing sintering. The thermoelectric properties of ZrCo_1-x_Pd_x_Bi samples were analyzed and discussed. The results showed that the electrical properties of ZrCo_1-x_Pd_x_Bi, including electrical conductivity and the Seebeck coefficient, increase due to the substitution of Pd on Co site. The lattice thermal conductivity of ZrCo_1-x_Pd_x_Bi is markedly decreased because of the Pd/Co substitution. A minimum *κ*_L_ of 5.0 W/mK for ZrCo_0.91_Pd_0.09_Bi is achieved at 800 K. The figure of merit of ZrCo_1-x_Pd_x_Bi is boosted due to the depressed lattice thermal conductivity and the improved power factor. The highest value of figure of merit reaches 0.23 for ZrCo_0.97_Pd_0.03_Bi half-Heusler compound at 800 K.

## 1. Introduction

Due to the large-scale utilization of fossil fuel energy, humans are facing a worldwide energy crisis and environmental problem. Developing reliable and renewable energy technology is becoming an inevitable choice for mankind in the 21st century to deal with the energy crisis and achieve sustainable economic development. Thermoelectric materials have huge potential and broad prospects in the application of solid-state cooling, heat pump and waste heat recovery because they can realize direct conversion between electricity and heat. Generally, the efficiency of thermoelectric materials is determined by the dimensionless figure-of-merit, *ZT* = *σS*^2^*T*/(*κ*_e_+*κ*_L_), where *σ* is the electrical conductivity, *S* is the Seebeck coefficient, *σS*^2^ is the power factor (PF), *κ*_e_ and *κ*_L_ are the respective electronic thermal conductivity and lattice thermal conductivity of total thermal conductivity *κ* [1,2,3]. The *ZT* of thermoelectric material can be enhanced by increasing the power factor and/or by decreasing the thermal conductivity. However, the complex interdependency of *S*, *σ*, and *κ*_e_ makes it difficult to boost the figure of merit by independently adjusting a parameter. Many advances in *ZT* of thermoelectric materials have been obtained by all-scale phono scattering, point defect, nanocomposite, the carrier concentration optimization and band engineering, et al. [4,5,6]. 

Half-Heusler (HH) compounds with cubic MgAgAs structure (F-43m) have been extensively studied due to their promising properties such as high mechanical strength and reliability, non-toxicity et al. HH compounds with 18 valence electrons per unit cell such as (Hf, Ti, Zr)CoSb and(Hf, Zr, Ti)NiS, exhibit unusual thermoelectric performance due to the sharp slope of their density of states (DOS) near Fermi level and narrow gap [7,8,9]. One of the key issues of HH compounds is the relatively high *κ*_L_. Many efforts focusing on the decrease of *κ* have been performed in order to enhance the *ZT* of HH alloys. The substitution of each site in cubic MgAgAs structure is a useful approach to enhance the thermoelectric properties of HH alloys because the position of Fermi energy can be adjusted and the *κ*_L_ can be decreased by alloy scattering. Tang et al. found interstitial Ni atoms in TiNiSn can scatter the phonons markedly and decrease the *κ*_L_, which results in the enhancement of *ZT* for Ni-rich TiNiSn compounds [10]. Huang et al. reported that Sn substitution leads to a doubled *S* and decreases the *κ*_e_ of NbCoSb_1-x_Sn_x_; thus, a maximal figure of merit of 0.54 is obtained [11]. Uher et al. and Rausch et al. reported that the mass disorder in the (Ti, Zr, Hf)-site lattice can result in extra phonon scattering, therefore decreasing the *κ* of (Hf, Zr, Ti)CoSb and (Hf, Zr)NiSn [12,13]. A good deal of data about the thermoelectric properties of *n*-type (Hf, Zr, Ti)CoSb and (Hf, Zr, Ti)NiSn [14,15,16] have been reported. However, there is little information about *n*-type ZrCoBi compounds.

In this work, the *n*-type Pd-doped ZrCo_1-x_Pd_x_Bi half-Heusler alloys were synthesized by arc-melting and rapid hot-pressing sintering. Thermoelectric properties of Pd-doped ZrCo_1-x_Pd_x_Bi half-Heusler alloys were measured and discussed. To our knowledge, this is the first report on the study about the ZrCo_1-x_Pd_x_Bi half-Heusler compound. We hoped that the Pd/Co substitution could enhance the *S*^2^*σ* and decrease the *κ*_L_ and then boost the figure of merit. 

## 2. Experimental Procedures

**Figure 1:**
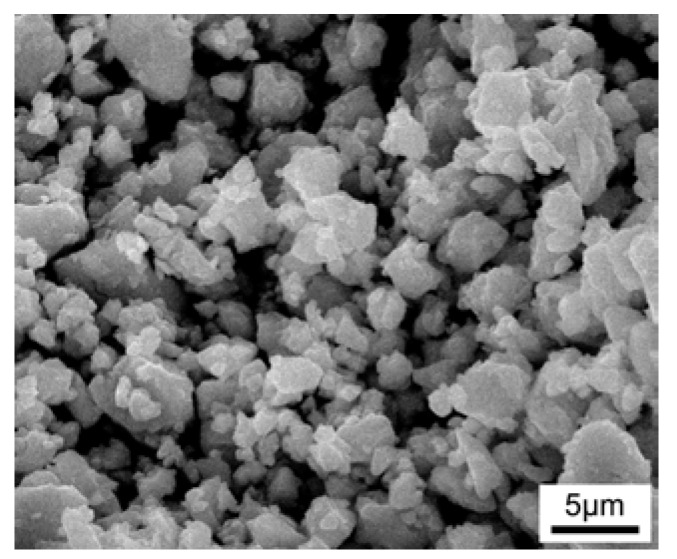
SEM of as-milled ZrCo_0.97_Pd_0.03_Bi half-Heusler (HH) powder.

The ingots with nominal composition ZrCo_1-x_Pd_x_Bi (*x* = 0, 0.03, 0.06, 0.09) were synthesized by arc-melting stoichiometric amounts of Bi (rod, 99.98%), Pd (granule, 99.999%), Co (granule, 99.999%), and Zr (slug, 99.98%) under an argon atmosphere. Then, the resultant ingots were ground into fine powders in agate mortar. After pulverizing in agate mortar, the powder of ZrCo_1-x_Pd_x_Bi was ball-milled in in a planetary ball milling machine using zirconia balls at 180 rpm for 10 h. Then the resulting powder was sieved using a 400-mesh sieve before hot consolidation. The average size of the obtained ZrCo_1-x_Pd_x_Bi powder was about 3.5 µm, as shown in Figure 1. The pulverized powders were consolidated by a rapid hot-press sintering process at 1323 K under the axial pressure of 65 MPa for 8 min in vacuum. The rapid hot-pressing sintering process was carried out in self-made equipment. The heating rate of rapid hot-pressing sintering is about 100 °C/min. The densities (*ρ*) of sintered ZrCo_1-x_Pd_x_Bi samples were measured by Archimedes method. The constituent phases and microstructure of ZrCo_1-x_Pd_x_Bi were characterized by X-ray diffraction (XRD, Siemens D5000, Bruker, Billerica, MA, USA) and field-emission scanning electron microscopy (FE-SEM, JXA-8200, JEOL, Tokyo, Japan), respectively. The electrical transport property of ZrCo_1-x_Pd_x_Bi samples including Seebeck coefficient (*S*) and electrical conductivity (*σ*) was measured by ZEM-3 instrument (ULVAC-RIKO, Yokohama, Japan) in helium. The total thermal conductivity of ZrCo_1-x_Pd_x_Bi was calculated using *κ* = *DρC_p_*, where *D* is thermal diffusion coefficient, *ρ* is the density, and *C_p_* is the specific heat capacity. D was obtained using the laser flash method (ULVAC-RIKO, TC7000) under argon. The specific heat capacity was determined using a differential scanning calorimetry (Perkin-Elmer, Waltham, MA, USA). The Hall coefficient (*R*_H_) measurements of ZrCo_1-x_Pd_x_Bi samples at room temperature were carried out by a Hall effect system in vacuum under a varied magnetic field ranging from −0.5 T to +0.5 T. The carrier concentration (*n*) and Hall mobility (*µ*_H_) were calculated based on the Hall coefficient according to *n* = 1/(*eR*_H_) and *µ*_H_ = *R*_H_*σ*, respectively, where *e* is the electron charge.

## 3. Results and Discussion

**Figure 2:**
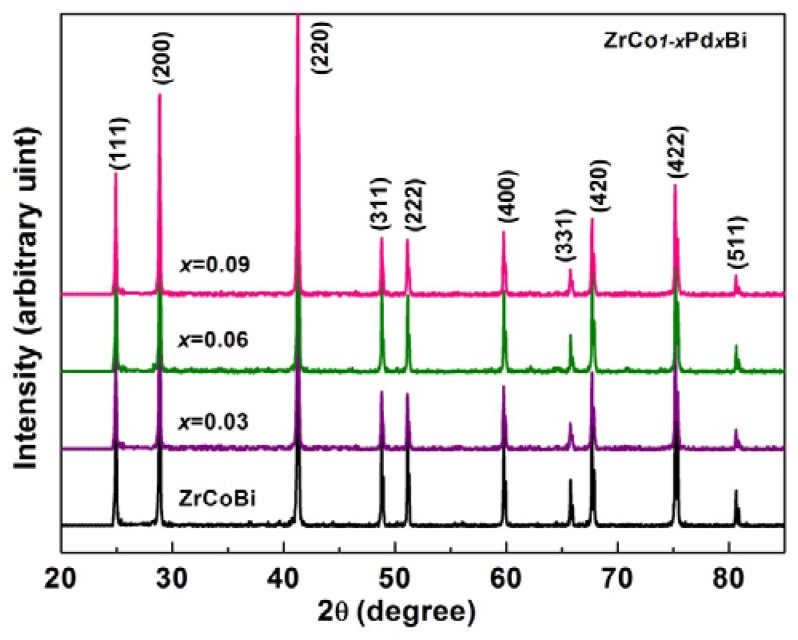
XRD diagrams of sintered ZrCo_1-x_Pd_x_Bi (*x* = 0, 0.03, 0.06, 0.09) HH samples.

**Figure 3:**
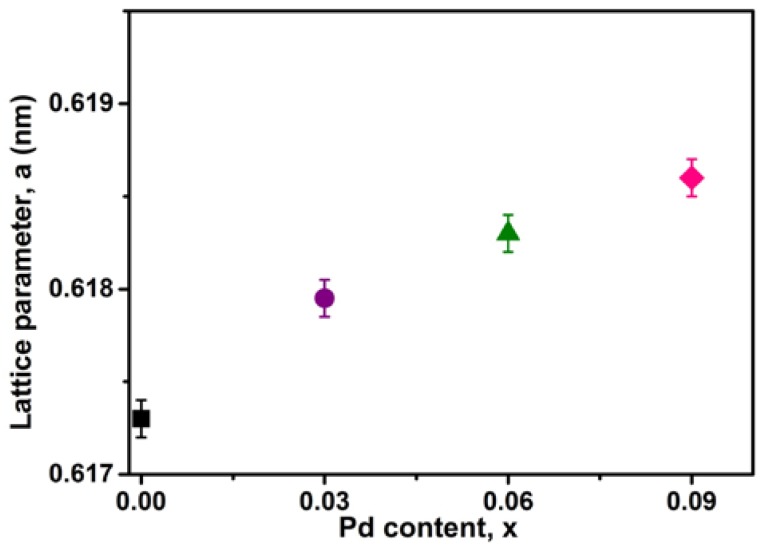
The lattice constant of ZrCo_1-x_Pd_x_Bi HH samples.

The XRD diagrams of the sintered bulk ZrCo_1-x_Pd_x_Bi samples are shown in Figure 2. All major diffraction peaks could be identified and indexed as the half-Heusler ZrCoBi phase (JCPDS 51-1255) without impurity phase under the detecting limit of XRD. The calculated lattice constant of ZrCoBi is 0.6174 nm, which is consistent with the previous report [17]. As shown in Figure 3, the calculated lattice constant of ZrCo_1-x_Pd_x_Bi (*x* = 0, 0.03, 0.0.06, 0.0.09) samples gradually increases with the Pd-doping content increasing, which is attributed to the bigger atom radius of Pd than Co. The lattice constant and error evaluation were calculated according to the references [18,19,20]. The relative densities of ZrCo_1-x_Pd_x_Bi samples were calculated and are listed in Table 1, high relative densities can ensure the measurement of thermoelectric properties.

**Figure 4:**
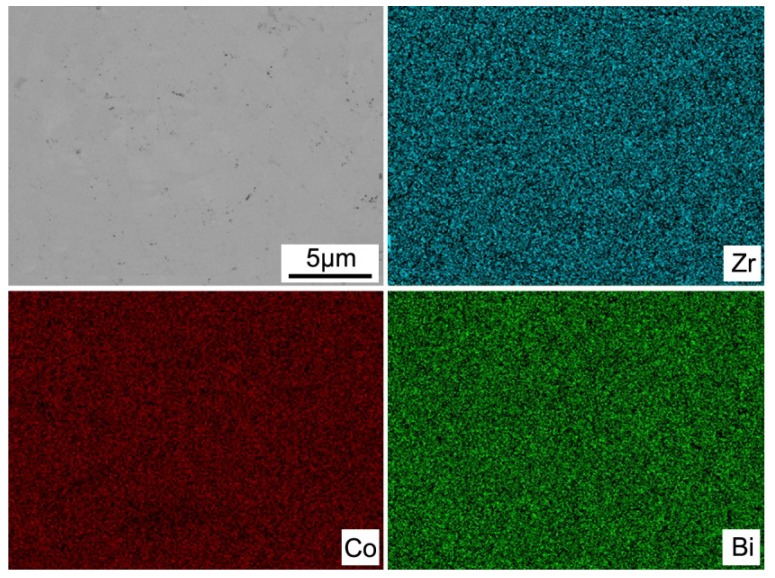
Backscattered electron SEM and elemental distribution of ZrCo_0.97_Pd_0.03_Bi HH samples.

**Figure 5:**
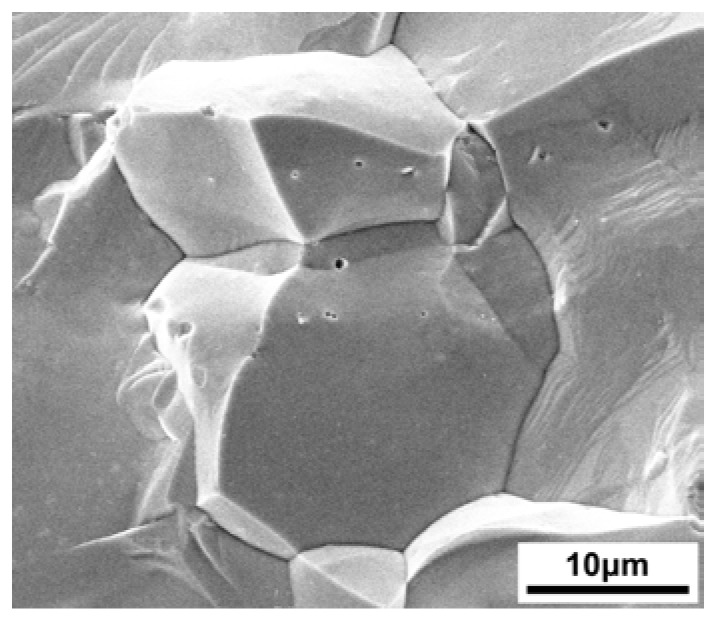
The fractural SEM image of the sintered ZrCo_0.97_Pd_0.03_Bi HH samples.

**Figure 6:**
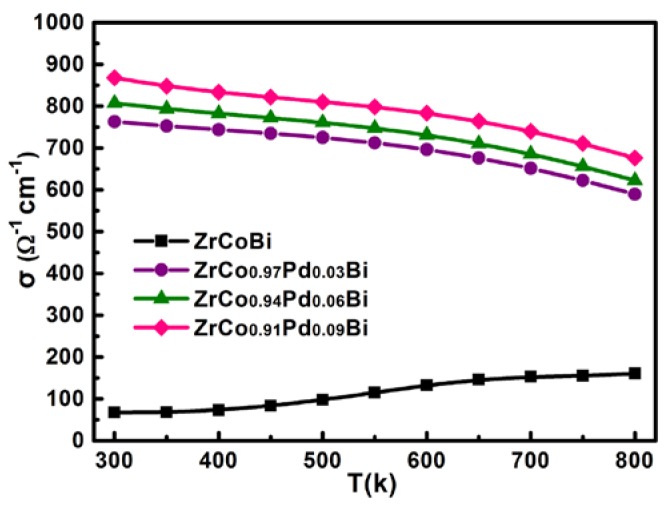
The variation of electrical conductivity for ZrCo_1-x_Pd_x_Bi HH samples.

Figure 4 displays the SEM image and the elemental distribution of the sintered ZrCo_0.97_Pd_0.03_Bi HH compound. It can be clearly noted from the images that all elements were distributed homogeneously and no evident other phase or crack existed, suggesting the ZrCo_0.97_Pd_0.03_Bi HH sample is the single phase that is in agreement with the XRD results. In addition, the fractural SEM showed that the grain size of sintered bulk ZrCo_0.97_Pd_0.03_Bi half-Heusler samples is about 15 µm, just as shown in Figure 5. The variation of *σ* for ZrCo_1-x_Pd_x_Bi HH compounds is present in Figure 6. The ZrCoBi sample exhibits a typical semiconductor behavior and has the lowest *σ* value over the entire measurement range. However, the *σ* of Pd-doped ZrCo_1-x_Pd_x_Bi HH compounds shows the semi-metal behavior within the measured temperature range. Compared with the *σ* of pristine ZrCoBi sample, the *σ* of Pd-doped ZrCo_1-x_Pd_x_Bi samples increases with the Pd-doping content rising. As Pd can donate more electrons than Co, the *n* of ZrCo_1-x_Pd_x_Bi samples is high than that of pristine ZrCoBi sample which results in the higher *σ*, just as shown in Table 1. For instance, the *n* of ZrCo_0.91_Pd_0.09_Bi sample increases to 5.25 × 10^20^ cm^−3^. 

**Figure 7:**
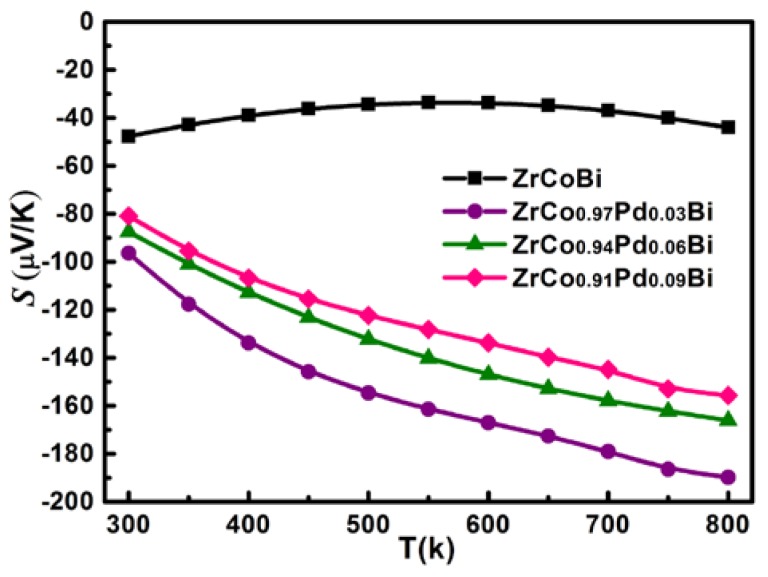
The variation of Seebeck coefficient for ZrCo_1-x_Pd_x_Bi HH samples.

**Figure 8:**
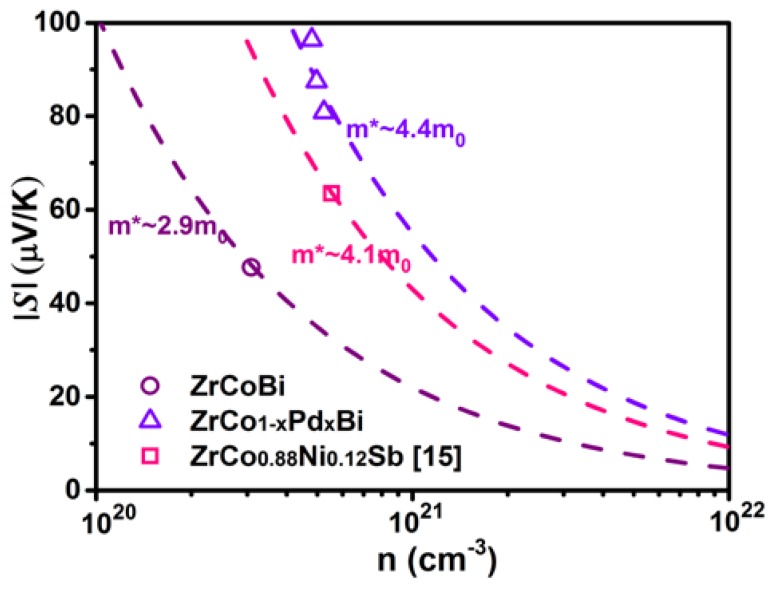
Carrier concentration dependence of Seebeck coefficient (Pisarenko) plots for ZrCo_1-x_Pd_x_Bi.

**Figure 9:**
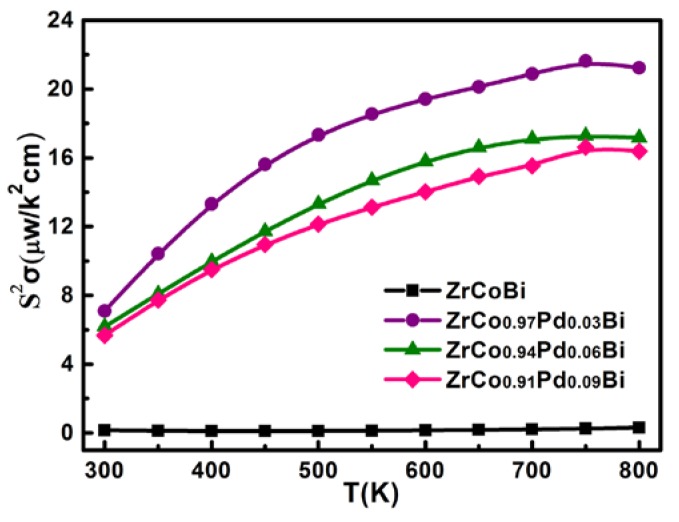
The variation of power factor for ZrCo_1-x_Pd_x_Bi HH samples.

Figure 7 shows the variation of *S* for ZrCo_1-x_Pd_x_Bi HH samples. All ZrCo_1-x_Pd_x_Bi samples have negative *S* and display the *n*-type conduction. The absolute value of *S* for ZrCo_1-x_Pd_x_Bi samples first increases and then decreases with the Pd-doping content rising. The *S* of ZrCo_0.97_Pd_0.03_Bi reaches a maximum of −191 µV/K at 800 K. In addition, the linear increase of *S* with rising temperature indicates that the ZrCo_1-x_Pd_x_Bi samples have single band conduction behavior. The Pd-doping could influence the band structure, which maybe increases the DOS near the Fermi level leading to the increase of *S*. Figure 8 shows the Pisarenko plots at 300 K of ZrCo_1-x_Pd_x_Bi samples which were derived by the relation (1)–(3) based on the single parabolic band (SPB) model [21]:

(1) $$LS=\pm \frac{k_{B}}{e}\left[\xi -\frac{\left(r+5/2\right)F_{r+3/2}\left(\xi \right)}{\left(r+3/2\right)F_{r+1/2}\left(\xi \right)}\right],$$

(2) $$F_{n}\left(\xi \right)=\int_{0}^{\infty }\frac{x^{n}}{1+e^{x-\xi }}dx,$$

(3) $$n=4\pi {\left(\frac{2m^{*}k_{B}T}{h^{2}}\right)}^{3/2}\times F_{1/2}\left(\xi \right),$$

where *h*, *k*_B_, *r*, *m*^∗^, and *ξ* are Planck’s constant, Boltzmann constant, scattering factor, the DOS effective mass and the reduced Fermi energy, respectively. The DOS effective mass of Pd-doped ZrCo_1-x_Pd_x_Bi samples was about 4.4m_0_, which is higher than that of pristine ZrCoBi (2.9m_0_). Therefore, the *S* of Pd-doped ZrCo_1-x_Pd_x_Bi samples is enhanced. According to the *σ* and *S*, the PF of ZrCo_1-x_Pd_x_Bi compounds are calculated in Figure 9. It can be seen that the PF of ZrCo_1-x_Pd_x_Bi compounds is markedly improved. The power factor of ZrCo_0.97_Pd_0.03_Bi reaches 21 μW·K^−2^·cm^−1^ at 800 K, which is much higher than that of pristine ZrCoBi. 

**Figure 10:**
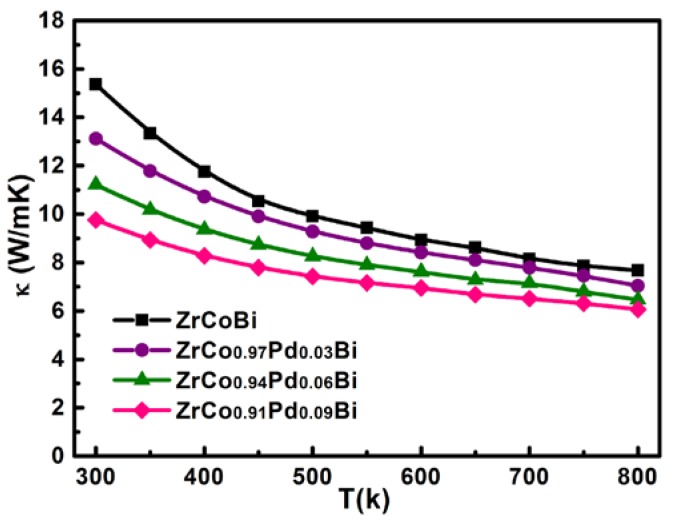
The variation of total thermal conductivity for ZrCo_1-x_Pd_x_Bi HH samples.

**Figure 11:**
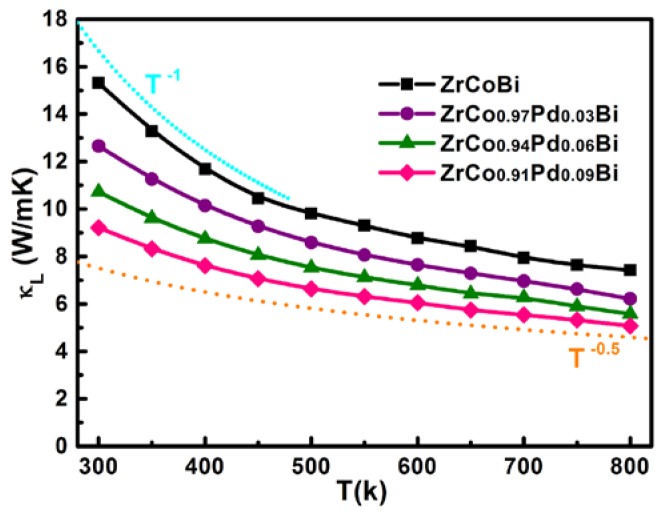
The variation of lattice thermal conductivity for ZrCo_1-x_Pd_x_Bi samples.

Figure 10 shows the variation of *κ* for ZrCo_1-x_Pd_x_Bi half-Heusler compounds within the whole measurement range. It can be observed that the *κ* of all ZrCo_1-x_Pd_x_Bi samples decreases with the temperature rising over the entire measured temperature due to the umklapp process. In addition, no bipolar effect is found in ZrCo_1-x_Pd_x_Bi samples. The *κ* of ZrCo_1-x_Pd_x_Bi samples decreases with the Pd-doping content rising which should be mainly due to the reduced *κ*_L_, just as shown in Figure 11. Generally, the *κ*_L_ can be obtained by directly extracting the *κ*_e_ from the total *κ* and *κ*_e_ can be estimated from the Wiedemann–Franz relation, *κ*_e_ = *LσT*, where *L* is the Lorenz number. *L* can be estimated by the respective *S* using the SPB model according to the relation (1)–(4) [22].

(4) $$L={\left(\frac{k_{B}}{e}\right)}^{2}\left[\frac{\left(r+7/2\right)F_{r+5/2}\left(\xi \right)}{\left(r+3/2\right)F_{r+1/2}\left(\xi \right)}-{\left(\frac{\left(r+5/2\right)F_{r+3/2}\left(\xi \right)}{\left(r+3/2\right)F_{r+1/2}\left(\xi \right)}\right)}^{2}\right].$$

The calculated Lorenz number at room temperature of pristine ZrCoBi half-Heusler is about 2.3 × 10^−8^ WΩ/K^2^ and with the temperature increasing, the Lorenz number decreases. However, the calculated Lorenz number of Pd-doped ZrCo_1-x_Pd_x_Bi half-Heusler compounds is lower than that of pristine ZrCoBi and in the range of (1.8–2.1) × 10^−8^ WΩ/K^2^.

**Figure 12:**
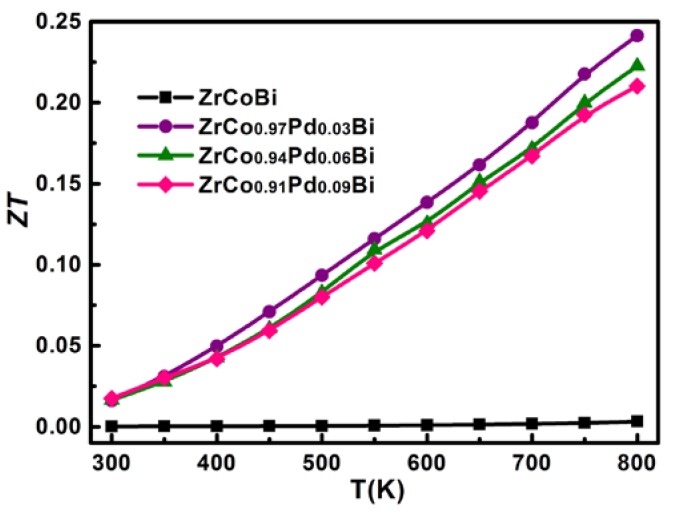
The variation of *ZT* for ZrCo_1-x_Pd_x_Bi half-Heusler samples.

As shown in Figure 11, the *κ*_L_ of ZrCo_1-x_Pd_x_Bi samples is markedly depressed because of the Pd/Co substitution. As the atom radius and mass of Pd are larger than those of Co, the Pd/Co substitution in the ZrCo_1-x_Pd_x_Bi samples could result in plenty of defect centers due to the strain field fluctuation (the differences of atom radius and interatomic coupling force) and the mass fluctuation (mass difference), which enhanced the phonon scattering [23]. Therefore, the *κ*_L_ of ZrCo_1-x_Pd_x_Bi samples decreases evidently. Generally, the lattice thermal conductivity can be estimated according to the relationship *κ*_L_ = 1/3*C_V_lV*, where *l* is the mean free path of phonon, *C_V_* is the heat capacity per unit volume and *V* is the average sound velocity. It is assumed the *C_V_* and *V* are constant. Then, the *κ*_L_ is determined by the mean free path *l*. The phonon scattering in materials usually has four mechanisms including phonon–phonon scattering (*l*∝T^−^^1^), point-defect scattering, grain boundary scattering (*l*∝grain size), and electron–phonon scattering (*l*∝T^−^^2^). The point-defect scattering is related with the mass and size disorder introduced by atomic substitution. At high temperature (above the Debye temperature), grain boundary scattering and electron–phonon scattering can be ignored without changing any physical trend. It can be noted that the *κ*_L_ of pristine ZrCoBi follows a T^−1^ relationship below 473 K and the temperature-dependence of T^−0.5^ above 473 K, indicating that the phonon–phonon scattering mechanism and the mixed scattering mechanism are the dominant scatterings, respectively. With the Pd-doping concentration rising, the *κ*_L_ of ZrCo_1-x_Pd_x_Bi samples deviates the T^−1^ and displays a lower slope which follows the T^−0.5^ behavior approximately, indicating the extra point defect scattering due to the Pd/Co substitution makes a large contribution to the phonon scattering [24,25]. The *κ*_L_ decreases from 15.4 W/mK for pristine ZrCoBi to 9.2 W/mK for ZrCo_0.91_Pd_0.09_Bi at room temperature and a minimum *κ*_L_ of 5.0 W/mK for ZrCo_0.91_Pd_0.09_Bi is obtained at 800 K. Figure 12 shows the temperature-dependent of *ZT* of ZrCo_1-x_Pd_x_Bi half-Heusler compounds. The *ZT* of pristine ZrCoBi half-Heusler is very low and almost has no change with the increase of temperature. Compared with the *ZT* of pristine of ZrCoBi half-Heusler, the *ZT* of Pd-doped ZrCo_1-x_Pd_x_Bi half-Heusler compounds at room temperature is evidently enhanced and is about 0.02. Moreover, the *ZT* values of Pd-doped ZrCo_1-x_Pd_x_Bi half-Heusler compounds increase with increasing temperature. Because of the improved power factor and the depressed *κ*_L_ by the Pd/Co substitution, the thermoelectric performance of ZrCo_1-x_Pd_x_Bi half-Heusler is boosted and the maximum figure of merit reaches 0.23 for ZrCo_0.97_Pd_0.03_Bi half-Heusler sample at 800 K.

## 4. Conclusions

The *n*-type Pd-doped ZrCo_1-x_Pd_x_Bi half-Heusler compounds were prepared by arc-melting and rapid hot-pressing sintering. By substituting Pd on Co sites, the power factor of ZrCo_1-x_Pd_x_Bi is obviously improved due to the increase in electrical conductivity and Seebeck coefficient. The lattice thermal conductivity of ZrCo_1-x_Pd_x_Bi is greatly decreased because of the Pd/Co substitution. A minimum *κ*_L_ of 5.0 W/mK for ZrCo_0.91_Pd_0.09_Bi is obtained at 800 K. The thermoelectric performance ZrCo_1-x_Pd_x_Bi half-Heusler is boosted due to the depressed lattice thermal conductivity and the improved power factor. The maximum figure of merit reaches 0.23 for ZrCo_0.97_Pd_0.03_Bi half-Heusler sample at 800 K.

## Figures and Tables

**Table 1:** The structural and carrier transport properties of ZrCo_1-x_Pd_x_Bi at room temperature.

*x*	Relative Density	*S* (μV/K)	*μ*_H_ (cm^2^·V^−1^·s^−1^)	*σ* (Ω^−1^·cm^−1^)	*κ*_L_ (W·m^−1^·K^−1^)	*n* (10^20^ cm^−3^)
0	98.1%	−47.7	1.37	67.6	16.2	3.09
0.03	98.6%	−96.4	9.94	762.8	14.2	4.80
0.06	97.9%	−87.5	10.1	807.2	12.7	4.97
0.09	98.8%	−80.9	10.3	867.7	10.3	5.25
